# Analysis of Mechanical Properties of Crumb Rubber Tires Mixed with Silty Sand of Various Sizes and Percentages

**DOI:** 10.3390/polym17152144

**Published:** 2025-08-05

**Authors:** Sindambiwe Theogene, Jianxiu Sun, Yanzi Wang, Run Xu, Jie Sun, Yuchen Tao, Changyong Zhang, Qingshuo Sun, Jiandong Wu, Hongya Yue, Hongbo Zhang

**Affiliations:** 1 School of Qilu Transportation, Shandong University, Jinan 250100, China; sindambiwetheos@gmail.com (S.T.); yanziwang@mail.sdu.edu.cn (Y.W.); 15957592560@163.com (Y.T.); 202415473@mail.sdu.edu.cn (Q.S.); yuehongya@126.com (H.Y.); 2 Shandong Provincial Communications Planning and Design Institute Co., Ltd., Jinan 250101, China; 19015122374@163.com (J.S.); xurun1@163.com (R.X.); cy_zhang@163.com (C.Z.); jdwu_mail@163.com (J.W.); 3 College of Transportation, Shandong University of Science and Technology, Qingdao 266510, China; ytyxlvyan@163.com

**Keywords:** crumb rubber tire mixed with silty sand, triaxial tests, deviatoric stress, stiffness modulus and shear stress

## Abstract

Every year, a billion tires are discarded worldwide, with only a small percentage being recycled. This leads to significant environmental hazards, such as fire risks and improper disposal. Silty sand also presents technical challenges due to its poor shear strength, susceptibility to erosion, and low permeability. This study explores the incorporation of crumb rubber derived from waste tires into silty sand to enhance its mechanical properties. Crumb rubber particles of varying sizes (3–6 mm, 5–10 mm, and 10–20 mm) were mixed with silty sand at 0%, 3%, 6%, and 9% percentages, respectively. Triaxial compression tests of unconsolidated and consolidated undrained tests with cell pressures of 100, 300, and 500 kPa were conducted. The deviatoric stress, shear stress, and stiffness modulus were investigated. The results revealed that the addition of crumb rubber significantly increased the deviatoric and shear stresses, especially at particle sizes of 5–10 mm, with contents of 3%, 6%, and 9%. Additionally, the stiffness modulus was notably reduced in the mixture containing 6% crumb rubber tire. These findings suggest that incorporating crumb rubber tires into silty sand not only improves silty sand performance but also offers an environmentally sustainable approach to tire waste recycling, making it a viable strategy for silty sand stabilization in construction and geotechnical engineering performance.

## 1. Introduction

Every year, 1.5 billion tires are wasted, and 3 billion new tires are produced worldwide; recycled tires make up 4% of the garbage. Road banks, city borders, dumping sites, and wetlands are among the places where used tires are improperly disposed of. The number of tires being stored continues to rise due to the growing demand for tires in various parts of the world [1,2]. Abandoned rubber tires pose a significant fire hazard; therefore, finding new uses for them or safe disposal methods is becoming increasingly important. Teams of geotechnical experts have proposed several uses for discarded tires, such as backfilling for retaining structures and as a filler in sidewalks and other pathways [3].

Humans have fortified their natural soils throughout history. It was well recognized before the Christian era that some areas had difficult natural conditions that made it hard for people to move between towns and villages. Both the Romans and Mesopotamians reported that weak soils may be strengthened by the addition of stabilizing substances such as calcium or crushed limestone [4]. In one instance, by naturally incorporating fiber inclusions that are oriented randomly, plant roots have contributed significantly to the stabilization of natural slopes and the improvement of soil strength. By strengthening the soil structure, this procedure increases stability and erosion resistance [5,6]. In the contemporary history of soil stabilization, Vidal is recognized for having invented the idea and principle of soil reinforcement. His pioneering research revealed that adding reinforcing components such as materials or structures to a soil mass greatly increases the shear resistance of the medium. This realization has greatly influenced soil engineering and has played a key role in the development of numerous soil stabilization technologies [7,8].

The absorption of waste tire materials, especially crumb rubber tires, into the soil has recently attracted attention as a way of improving soil quality over the long term and reducing the environmental risk related to tire disposal [9,10]. This technique is frequently used in civil engineering and building projects since it is successful in increasing the strength, stability, and durability of soil [11]. Furthermore, using discarded tire materials in geotechnical engineering applications lessens the environmental impact of tire disposal and supports sustainable waste management methods [12].

Recently, silty sand has become a significant worldwide issue that requires all-encompassing geotechnical solutions. Its intricate behavior is crucial in tackling urgent problems such as improving urban resilience and sustainability and effectively managing material and energy resources. To address these issues, basic research is needed to characterize subsurface conditions better, monitor complex geomaterial behaviors, and manage geotechnical and environmental systems that are multiscale and multi-process. To make more precise forecasts and make well-informed decisions in increasingly complex situations, there is an increasing need to incorporate data science and machine learning techniques into geotechnical engineering research and practice [13].

Silty sand is a typical soil type that can be found in many places. In light of its intrinsically weak mechanical characteristics, such as low shear strength, poor drainage capacity, high compressibility, and a propensity to erode easily, silty sand poses serious geotechnical challenges. These features restrict its applicability for direct use in crucial infrastructure projects where long-term stability is crucial, such as retaining walls, road subgrades, and embankments. Because silty sand is fine-grained, it can hold water and create excess pore water pressure, especially when subjected to cyclic or continuous loading. This can lead to instability, settlement, or even liquefaction. On the other hand, clean sands or gravels, which are well-graded granular soils, provide superior shear strength and drainage. Thus, stabilizing or reinforcing silty sand to improve its engineering performance is essential for ensuring safe and sustainable construction [14].

The integration of waste materials into silty sand for geotechnical applications has been a subject of interest for engineers and researchers seeking sustainable solutions to address environmental challenges and improve the performance of the civil infrastructure [15,16] The exceptional elasticity, robustness, and longevity of crumb rubber are among the special qualities that enhance the functionality of reinforced soil structures [17]. Furthermore, studies have investigated how different elements, including the size, quantity, and distribution of rubber particles, affect the mechanical properties of reinforced silty sand, offering insights into the best mix designs [18].

Additionally, a thorough understanding of how the mechanical characteristics of this composite material affect its behavior is currently lacking. This study intends to bridge this knowledge gap and advance our understanding of how silty sand behaves with crumb tires and how it can be used in geotechnical engineering to support environmentally friendly infrastructure and environmentally responsible development by thoroughly analyzing silty sand reinforced with various percentages of crumb tires.

## 2. Review of the Risk of Recycling Crumb Rubber Tires

Although evaluating the possible health or water conservation risks of recycling crumb rubber is not the main goal of this study, a brief review was included to shed more light on the advantages of using it as well as any possible environmental or health issues.

Research published by John Fort et al. revealed that an increased concentration of zinc was observed in the analyzed rubber crumb, which may pose a risk to aquatic organisms in particular [19]. On the other hand, relatively low concentrations of other heavy metals were detected, which, according to the available literature, do not pose a danger to the human body. Considering the studies aimed directly at crumb rubber assessing the Human Health Harm Assessment (HHRA), the results revealed that the growing amount of data present little harm to human health when recycled rubber infill is utilized in synthetic turf [20,21]. This assessment provides useful information to help stakeholders engage in the recycling of crumb tires.

## 3. Materials and Methods

This research was conducted to investigate the behavior of silty sand and crumb tires; two different types of test samples were examined. First, there was unreinforced silty sand; second, CRT was mixed with silty sand at 3%, 6%, and 9% crumb tires, and the sizes were 3–6 mm, 5–10 mm, and 10–20 mm, respectively.

### 3.1. Materials

#### 3.1.1. Sieve Test Description for Silty Sand

**Figure 1:**
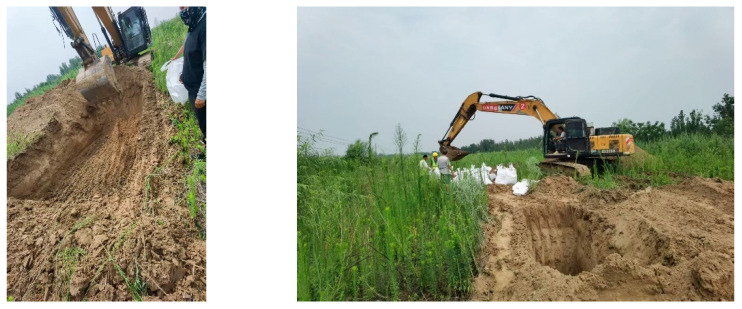
Silty sand extraction quarry.

The silt sand used in this research was extracted near the Yellow River, Dawangmiao village, Daqiao Street, Tianqiao District, Shandong Province, China. Figure 1 present the extraction of silty sand quarry location.

Considering each policy code when classifying soils did not significantly alter the majority of methods, such as the ISSCS (Indian Standard Soil Classification System), IS 1498, AASHTO (American Association of State Highway and Transportation Officials), and the USCS (Unified Soil Classification System) standardized in ASTM D2487 [22,23].

A sample of silty sand was prepared in a geotechnical laboratory and drawn into successively larger sieves from top to bottom to ascertain the grain size partition of the soil. The proportion of the silty sand sample that passed through each sieve was recorded [24,25,26,27].

**Figure 2:**
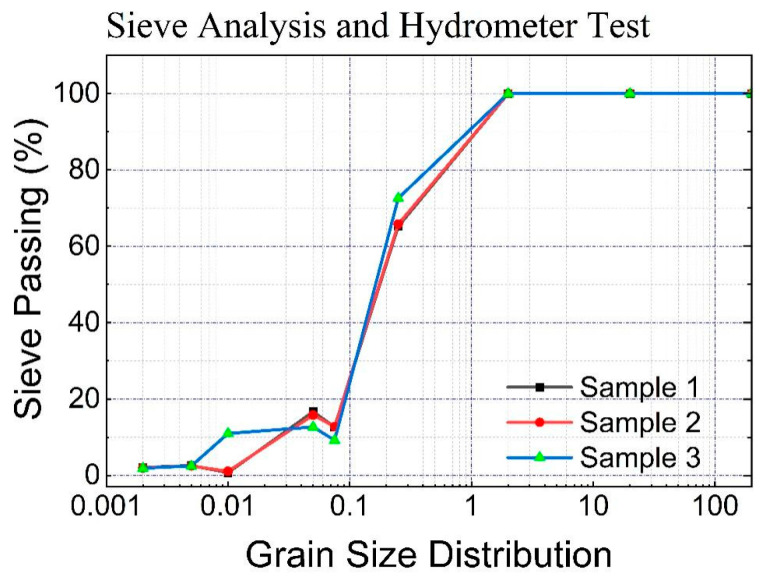
Sieve analysis and hydrometer test.

To essentially determine the soil classification, the China Code (GB 50021-2001) was used, and three samples of silty sand were sieved to determine the passing weight of each sieve from the highest to the lowest size, as basically recorded (refer to Figure 2). The samples noted as 1 and 2 are closely correlated. This means that the results obtained are closely similar to those of the samples in series 3. The results are clearly greater than those of the last two test results, even if there is no large difference between them. In general, the average number of samples passing through sieves of 0.25 mm to 0.075 mm, particularly the average, is 67.9%, and the number of samples retained in the hydrometer test from 0.075 mm to 0.005, which actually has an average of 11.56% from 0.005 to 0.002, is 2.53% below 0.002, with an average of 2.0%, which refers to silty sand among the soil classifications in China, and Table 1 illustrate the physical properties of silty sand.

##### Composition of Silty Sand

Silty sand is a soil type made up mostly of sand particles (coarse-grained, 0.075–4.75 mm) and a significant fraction of silty particles (fine-grained, less than 0.075 mm), as well as modest amounts of clay or organic materials, depending on its origin. It frequently contains minerals such as quartz, feldspar, mica, and carbonates. In the silty-sized particles, quartz becomes the predominant mineral; in clay-sized fractions, it becomes a minor component. At finer scales, mineral stability and particle breakup have an impact on this change. The quantity and orientation of microfracture sets in the source rock largely dictate the shape of the quartz grains. The grains’ form and texture are determined by these cracks. Importantly, rather than being influenced by sedimentary environments, the surface characteristics of the majority of quartz grains are the product of pre-sedimentary geological processes, reflecting the crystallization and deformation history of the source rock [28]. Table 2 shows the chemical structure of the silty sand.

Because of its heterogeneous mineralogical makeup, silty sand lacks a single, consistent chemical formula. Instead, it is a heterogeneous mixture of silicate-based minerals largely composed of silicon (Si), aluminum (Al), and oxygen (O), with minor amounts of iron (Fe), potassium (K), magnesium (Mg), and sodium (Na).

#### 3.1.2. Crumb Rubber Tires

The crumb rubber tire used was purchased from an online shop (Alibaba), and the address location was Hushan village, Qingyang, Zouping County, Shandong Province, China.

**Figure 3:**
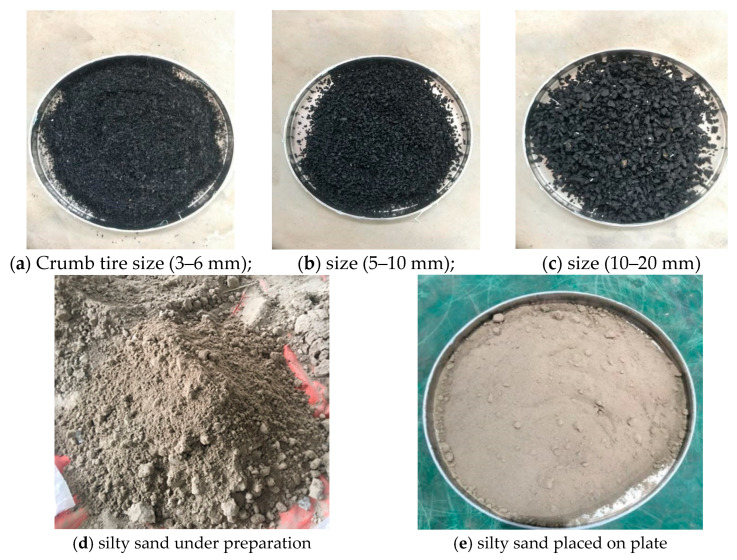
Crumb rubber tire (CRT) particle sizes of 3–6, 5–10 and 10–20 mm and silty sand to be mixed together.

Tire waste is categorized according to several criteria, including composition, source, and condition. For efficient waste management and recycling procedures, waste tires must be classified [29]. The results show that civil engineering has been the focus of many studies and inquiries. Given the crucial role that civil engineering plays in the development of infrastructure, construction, and urban planning, it indicates that there is a great deal of interest in this field of application [30]. In this study, rubber granulate (0.8–20 mm) was included in the categories of rubber recycling materials identified by CEN TS14243 [31] and from Agreement 14243–2002 of the CEN Workshop [32]. Importantly, different enterprises may classify tires differently. This implies that there may be variations in the classification of discarded tires. Figure 3 shows the crumb rubber size.

##### Composition of Crumb Rubber Tires

Crumb rubber is made by mechanically grinding old automobile tires into small granules. It is made up of a combination of natural rubber (polyisoprene) and synthetic rubber, predominantly styrene-butadiene rubber (SBR), as well as numerous other materials. Carbon black serves as a reinforcing filler, as do sulfur and chemical accelerators used in the vulcanization process, aromatic or paraffinic processing oils, and different resins, antioxidants, and plasticizers. Depending on how thorough the processing is, trace amounts of steel and textile fibers may be left behind [33]. Table 3 below describes the chemical structure of crumb tire.

During vulcanization, sulfur atoms create cross-links (-S- or -S-S-) between rubber polymer chains. These sulfur bridges form a three-dimensional network, increasing rubber elasticity, heat resistance, and overall durability.

### 3.2. Methods

Thirty-three triaxial tests were carried out, the total volume of mold was determined, and a filled weight was calculated using the density of silty sand, CRT mixed with silty sand, at weight percentages of 0%, 3%, 6%, and 9% for triaxial compression testing (see Table 4 and Table 5). The soil mixtures were manually compacted, and the total weight of each mixture was divided into three equal sections to achieve equal density. Each layer was compressed within a mold via a heavy flat-bottomed metal tamper. A tape measure was employed to ensure that each compacted layer did not exceed 10 cm in height, hence preserving consistency among samples. The rigorous compaction technique reduced the amount of air space and ensured homogeneity throughout the sample. The compressed samples were then placed in the triaxial test device for examination. The experiments were run at a strain rate of 0.6 mm per minute with cell pressures of 100 kPa, 300 kPa, and 500 kPa, for unconsolidated–undrained (UU) samples (see Table 4), and Consolidated–undrained (CU) (see Table 5).

In this study, crumb rubber tire contents with particularly excellent mechanical properties were chosen for in-depth analysis via unconsolidated–undrained (UU) testing. Consolidated–undrained (CU) triaxial tests were then used in this study to provide a thorough and in-depth examination.

### 3.3. Sample Preparation

In this investigation, silty sand was reinforced with crumb rubber tires, which are a sustainable and lightweight material. The Alibaba online marketplace in China was used to purchase crumb rubber, which is located in Hushan Village, Qingyang, Zouping Country, Shandong Province. This acts as a marketplace that links customers and recycling businesses for used tires. To assess the impact of particle size on the mechanical behavior of the composite material, three distinct rubber particle size ranges of 3–6 mm, 5–10 mm, and 10–20 mm were chosen to represent a spectrum from fine to coarse granules.

**Figure 4:**
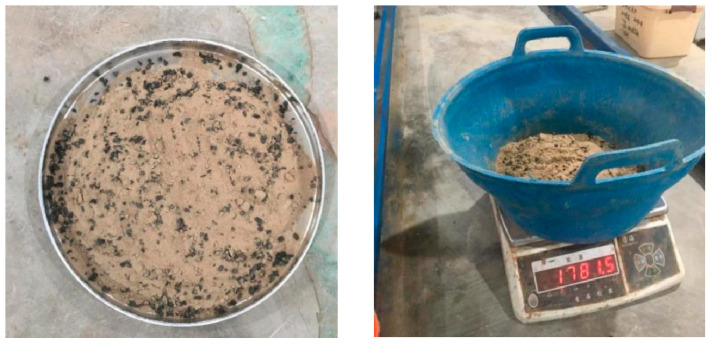
CRT mixed with silty sand weighted according to the proportion in Table 4 and Table 5.

The silty sand and crumb rubber were first collected and kept in a controlled environment to prepare the test samples. Each size group was chosen at random. After that, three distinct weight ratios of rubber to silty sand were combined: 3%, 6%, and 9% of the silty sand’s dry weight. Overall, nine distinct combinations were generated. To guarantee uniform distribution and prevent segregation, the mixing procedure was carried out by hand. The mixtures were prepared for laboratory testing and maintained appropriately after homogeneity was confirmed. Table 4 and Table 5 provide specifics about the mix proportions and particle size distributions, whereas Figure 4 depicts the material’s appearance and preparation procedure.

#### 3.3.1. Triaxial Test Procedure and Apparatus

##### Apparatus

**Figure 5:**
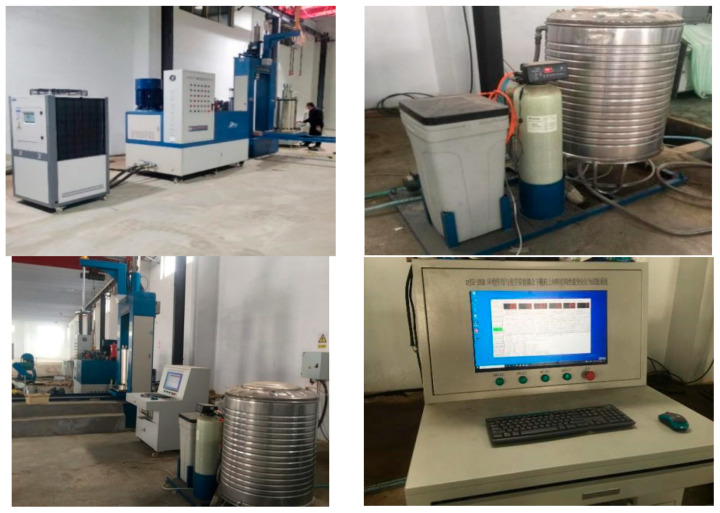
Triaxial test apparatus.

The triaxial test apparatus (see Figure 5) contains a generator to use in the event of an electrical outage, a system unit to control the entire triaxial process, a central processor unit (CPU) to display and monitor the triaxial operation, and a triaxial cell, which is a cylindrical container in which the soil sample is placed. A porous stone or filter at the base of the cell let the water out during testing while keeping the soil particles from passing through. The cylinders used in the testing had a diameter of 150 mm and a height of 300 mm.

##### Testing Procedure

**Figure 6:**
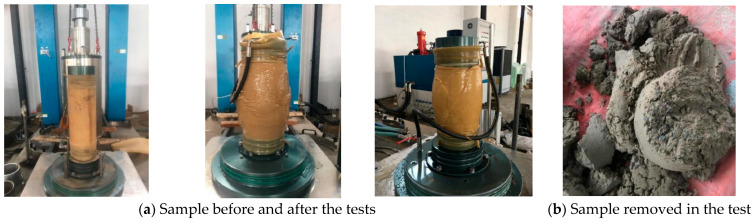
Samples processed at different times.

The triaxial test steps are as follows:

(1) The triaxial cell was first thoroughly cleaned, a rubber membrane was placed on the pedestal, the cylindrical container was positioned correctly, a water pipe was connected, an air pressure pipe was placed, and a membrane filler was placed on the pedestal’s bottom to prevent the sample from entering the hole.
(2) A silty sand sample mixture was weighed and compacted at three levels according to the cell height. Once all three layers had been filled and compacted, a second membrane filler was placed on top of the filled and compacted sample, and the pedestal was then positioned and secured on top. By opening valve number five, the air inside was removed. If the pressure on the monitor screen was greater than −60 kPa, it was turned off. This serves to hold the sample upright, and it works particularly well with granular materials that are not self-supporting.
(3) The cylindrical device that held the sample in place during compaction was removed, the small sample that had fallen was cleaned, and any issues found during compaction were noted for correction. The samples were then prepared for placement in a vacuum, which was released and fixed on the triaxial cell base. To prevent the loss of air pressure, the chamber should be secured by tightening the bolts, and then the system should proceed to the bottom load frame.
(4) The triaxial cell sample was moved on top and connected to the bottom of the loading frame via the monitor screen. The valve was open to fill the water vacuum; once it was full, the small outlet pipe connected to the top ran out of the water, and the pump and valve were closed. The sample was then subjected to pressure via a monitor screen to ensure that there was enough water and oil. The strain rate speed was set at 0.6 mm/minute at the start of the test.
(5) The name was set, and pressure was applied to the sample via the proper triaxial test procedure. When the pressure exceeded 100 kPa, valves 1 and 4 were opened to release the air pressure and waited until the applied pressure was reached. The axial force on the sample was applied, the shearing stage started, and the test continued until the sample reached failure [34,35,36]. Figure 6 show the sample processed after and before the triaxial test.

## 4. Test Results and Discussion

### 4.1. Triaxial Tests of Silty Sand and CRT Mixed with Silty Sand

#### 4.1.1. Sizes of 3–6 mm at 3%, 6%, and 9% Ratios

**Figure 7:**
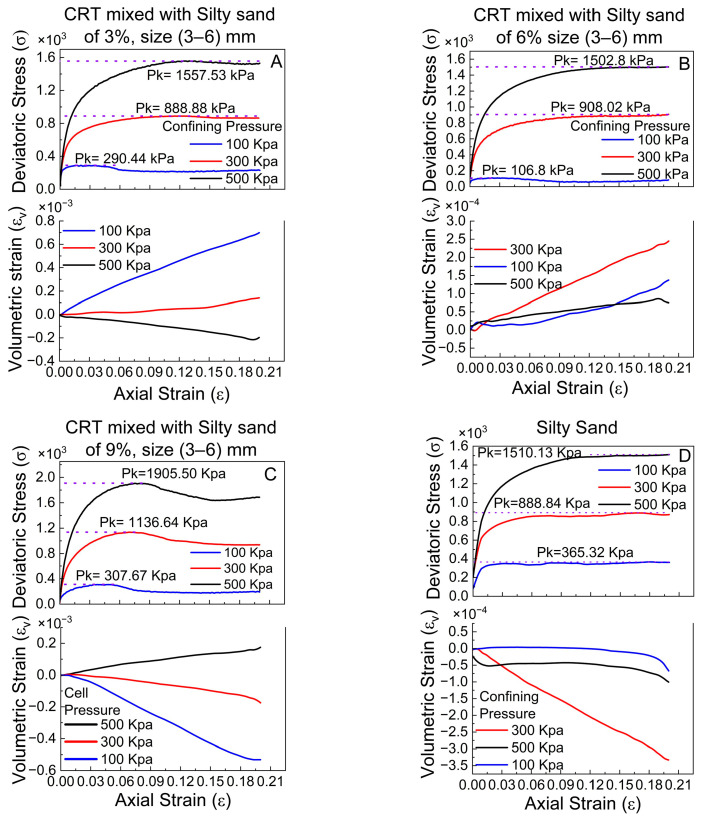
Deviatoric and volumetric stress results at confining pressure of 100, 300 and 500 Kpa of Triaxial tests of silty sand and CRT mixed with silty sand, with sizes of 3–6 mm at 3%, 6%, and 9%.

The graphs in Figure 7 present the results of unconsolidated–undrained triaxial compression tests on silty sand and CRT mixed with silty sand, with varying percentages of crumb tires (3%, 6%, and 9%) in the size range of 3–6 mm under different confining pressures (100 kPa, 300 kPa, and 500 kPa). The upper portion of each figure displays the deviatoric stress (σ) versus axial strain (ε), whereas the lower portion shows the volumetric strain (εv) against axial strain (ε). The three graphs show an increasing trend in peak deviatoric stress (Pk) as the crumb tire content increases, which suggests that higher reinforcement levels enhance the resistance of the material to deformation.

In Figure 7A, where the crumb tire content is 3%, at a confining pressure of 100 kPa, the deviator stress value peaks at 290.4 kPa at an axial strain of 0.03 and remains constant between 0.07 and 0.2. At an axial strain of 0.12, peak deviatoric stresses of 888.84 kPa and 1554.4 kPa were noted for confining pressures of 300 kPa and 500 kPa (see Figure 7A), respectively. The maximum deviatoric stress increases with increasing axial strain [37].

The volumetric strain under 100 kPa of confining pressure increases favorably as the axial strain increases from 0% to approximately 20%. Under 300 kPa of confining pressure, the volumetric strain remains constant between 0 and 0.03 axial strains before increasing with increasing axial strain. As the axial strain increases for the 500 kPa confining pressure, the volumetric strain decreases (Figure 7A). This result indicates a more significant volume change under lower confinement, which could be due to the compaction behavior of the silty sand mixed with crumb tires. As the crumb tire content increases, the material shows greater resistance to shear deformation.

The deviatoric stress increases to 902.5 kPa and 1502.4 kPa when a 6% crumb tire is added to the silty sand soil at confining pressures of 300 kPa and 500 kPa (Figure 7B), respectively. There are also ambiguous peaks at an axial strain of 0.2. It seems that the more tension there is, the more stress there is [37]. A favorable relationship between the volumetric strain and axial strain is demonstrated by plotting the volumetric strain against the axial strain for confining pressures of 100 kPa, 300 kPa, and 500 kPa.

The maximum deviatoric stresses for silty sand containing 9% crumb tire are 1134.64 kPa and 1905.50 kPa under confining pressures of 300 kPa and 500 kPa (Figure 7C), respectively, occurring at the same axial strain value of 0.075. At an axial strain of 0.03 and a confining pressure of 100 kPa, the maximum axial stress is 307.67 kPa. According to the graphs of the volumetric strain against axial strain, the volumetric strain increases with increasing axial strain at a confining pressure of 100 kPa and decreases with increasing axial strain at confining pressures of 300 and 500 kPa.

Similarly, the unreinforced silty sand (Figure 7D) has a deviatoric stress of 1510.13 kPa at an axial strain of 0.2 at a confining pressure of 500 kPa, 888.84 kPa at a confining pressure of 300 kPa, and 365.32 kPa deviatoric stress at an axial strain of 0.18 with a confining pressure of 100 kPa. The volumetric strain at a confining pressure of 100 kPa is constant at 0.18; at a confining pressure of 500 kPa, the strain varies, increases to 0.12, and then contracts until 0.2 axial strain is reached. At a confining pressure of 300 kPa, the volumetric strain contracts as the axial strain increases.

These observations highlight how the crumb tire content influences the material’s compressive and volumetric response, with higher percentages of crumb rubber tires generally leading to less volumetric change under applied pressures.

#### 4.1.2. Sizes of 5–10 mm at 3%, 6%, and 9% Ratios

**Figure 8:**
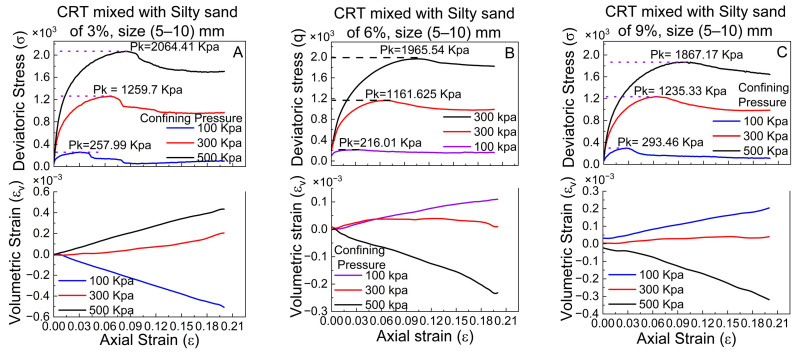
Deviatoric and volumetric stress results at confining pressure of 100, 300 and 500 kPa of CRT mixed with silty sand sizes of 5–10 mm at 3%, 6%, and 9% ratios.

An unconsolidated–undrained (UU) test was performed to investigate the behavior of silty sand combined with crumb tire percentages of 3%, 6%, and 9% with particle sizes ranging from 5 to 10 mm. The test findings, which are displayed in Figure 8, revealed clear increasing trends in peak deviatoric stress for each mixture at various confining pressures and axial strain levels.

The 3% crumb tire combination resulted in a peak deviatoric stress of 257.99 kPa at an axial strain of 0.03 when subjected to a confining pressure of 100 kPa (see Figure 8A). The highest deviatoric stress rose to 1259.7 kPa at an axial strain of 0.065 for a confining pressure of 300 kPa. The highest deviatoric stress increased to 2064.41 kPa at an axial strain of 0.084 at a confining pressure of 500 kPa. According to this observation (Figure 8A), the deviatoric stress increases with strain as the confining pressure increases. Volumetric strain analysis indicated that the volumetric strain decreased between 0 and 0.2 axial strains for the 3% crumb tire combination at a 100 kPa confining pressure. On the other hand, the volumetric strain increased in tandem with the axial strain for the tire compositions containing 6% and 9% crumb.

At 6% and 9% crumb, the peak deviatoric stress increases with increasing axial strain as the confining pressure increases. At a confining pressure of 500 kPa, the peak deviatoric stress decreases as the percentage of crumb tires increases (Figure 8A–C). At 300 kPa and 100 kPa, the deviatoric stresses at the 6% crumb tire are lower than 3% and 9% (see Figure 8B), respectively. The volumetric strain of reinforced silty sand with crumb tires of 6% and 9% at 100 kPa expands gradually with axial strain; at 500 kPa, it contracts at high levels, but at 300 kPa, it expands with axial strain at low levels, as expressed in Figure 8, except for the volumetric strain inversed at 500 kPa and 100 kPa of 3% crumb tires to 6% and 9%; at 300 kPa, all combinations of volumetric strains have the same trends.

A comparison of all the peak deviatoric stress findings of mixed reinforced silty sand revealed that the tire mixture containing 3% crumb produced the highest peak deviatoric stress of 2064.41 kPa (Figure 8A), which decreased with increasing tire content in the silty sand.

#### 4.1.3. Sizes of 10–20 mm at 3%, 6%, and 9% Ratios

**Figure 9:**
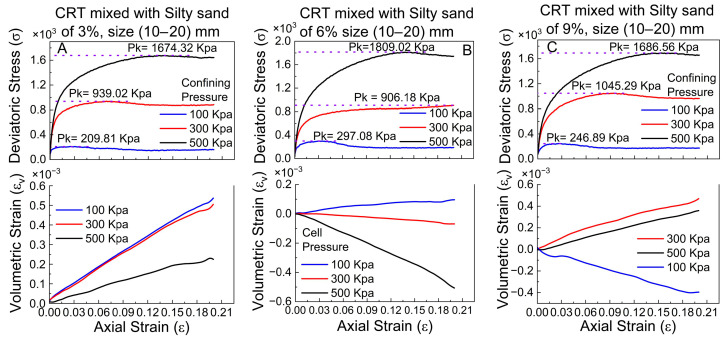
Deviatoric and volumetric stress results at confining pressure of 100, 300 and 500 kPa of CRT mixed with silty sand sizes of 10–20 mm at 3%, 6%, and 9% ratios.

Figure 9 depicts the correlation between deviatoric stress and axial strain, with the volumetric strain characteristics of silty sand mixed with different proportions (3%, 6%, and 9%) of crumb tire particle sizes ranging from 10 to 20 mm under varying, confining pressures (100 kPa, 300 kPa, and 500 kPa). In Figure 9A (3% crumb tire), the deviatoric stress increases swiftly with increasing axial strain until it stabilizes at high values of 209.81 kPa, 939.02 kPa, and 1674.32 kPa at confining pressures of 100 kPa, 300 kPa, and 500 kPa, respectively. The volumetric strain trends exhibit a steady increase with axial strain at 100 kPa and 300 kPa; however, at 500 kPa, the volumetric strain first increases before stabilizing, suggesting reduced compaction at elevated confinement levels.

In Figure 9B (6% crumb tire), the deviatoric stress patterns are analogous, albeit with marginally elevated peak values of 297.08 kPa, 906.18 kPa, and 1809.02 kPa for confining pressures of 100 kPa, 300 kPa, and 500 kPa, respectively. The volumetric strain behavior significantly changes with 3% crumb tire concentration; at 500 kPa, contraction (negative volumetric strain) occurs, indicating that densification results from the increased crumb tire content. At 100 kPa and 300 kPa, the volumetric strain demonstrates more stability with little change, reflecting the influence of increased crumb tire content on the compressibility of the material.

In Figure 9C (9% crumb tire), the deviatoric stress attains peak values of 246.89 kPa, 1045.29 kPa, and 1686.56 kPa at 100 kPa, 300 kPa, and 500 kPa, respectively. The maximum stress at 300 kPa exceeds that depicted in Figure 9B, indicating that a higher crumb tire composition improves the strength under mild confinement. The volumetric strain patterns indicate that at 100 kPa confinement, contraction follows an initial increase; however, at 300 kPa and 500 kPa, the volumetric strain continues to increase, albeit at a diminished pace. This behavior indicates that an increased crumb tire percentage enhances compressibility under lower confinement; however, at elevated confinement, the material experiences less volume change owing to particle interlocking effects. Finally, the maximum peak deviatoric stress for each of the three combinations increases with axial strain, and the confining pressure occurs at varying axial strain values.

### 4.2. Influence of Different Crumb Rubber Tire (CRT) Sizes

Table 4 shows the influence of various crumb rubber tire sizes (3–6, 5–10, and 10–20 mm) on the deviatoric stress of the soil mixtures subjected to various confining pressures. The size of the crumb rubber tire particles significantly affects the soil strength across all tire percentages (3%, 6%, and 9%). At a reduced confining pressure of 100 kPa, the particle size of the tire mixture has a diminished influence, with the 3% tire composition (regardless of size) exhibits comparatively low deviatoric stresses concerning silt–sand control. At elevated confining pressures (300 kPa and 500 kPa), the disparities among tire sizes become more pronounced, with medium-sized tire particles (5–10 mm) often exhibiting the highest deviatoric stresses across all percentages.

The 3% tire mixture exhibits the maximum deviatoric stress from 5 to 10 mm particles across all the confining pressures, particularly at 500 kPa (2064.41 kPa) (see Table 4). The subsequent size of 3–6 mm results in a notable increase in stress, with a value of 1557.53 kPa at 500 kPa. The largest tire size (10–20 mm) generates comparatively lower deviatoric stress, especially at 500 kPa (1674.32 kPa), indicating that the larger particles may not interlock as efficiently, or their increased surface area may lead to diminished reinforcement efficacy. This suggests that a medium tire size (5–10 mm) may offer optimal interparticle interactions and soil stabilization, especially under elevated confining pressures.

The tendency persists for the 6% and 9% tire combinations (see Table 4), with medium-sized tire particles (5–10 mm) yielding the highest deviatoric stresses at 500 kPa. The 6% tire mixture containing 5–10 mm particles yields a pressure of 1965.54 kPa, whereas the 9% tire mixture produces 1867.17 kPa, both exceeding the performance of the 3–6 mm and 10–20 mm tire sizes. The 9% tire combination containing 3–6 mm particles results in more stress (1905.5 kPa) than does the 9% mixture with 10–20 mm particles (1686.56 kPa). Overall, it is observed that medium tire sizes consistently yield the greatest stress under elevated confining pressures. The 10–20 mm tire particles, particularly in the 6% and 9% combinations, appear to provide a diminished level of augmentation, underscoring the significance of the tire particle size in improving the stress resistance of the soil under constrained conditions.

### 4.3. Influence of the CRT Mixing Ratio

**Figure 10:**
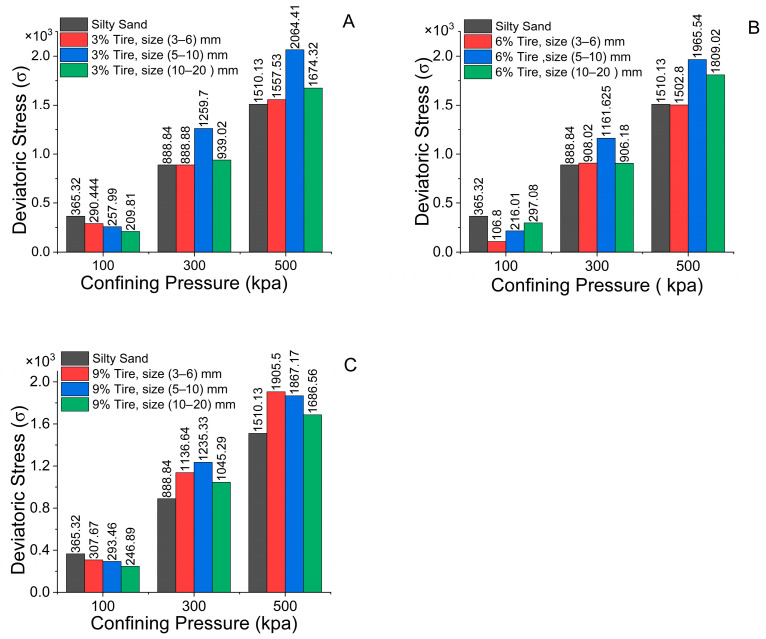
Comparison of the results of triaxial test considering the deviatoric stress at the same confining pressure, crumb rubber ratios, and different crumb tire sizes.

Table 6 shows the effects of various tire contents (3%, 6%, and 9%) and tire sizes (3–6 mm, 5–10 mm, and 10–20 mm) on the deviatoric stress at different confining pressures of 100 kPa, 300 kPa, and 500 kPa, respectively, for reinforced silty sand. The predominant pattern observed is an increase in deviatoric stress with increasing confining pressure across all combinations; however, the mixing ratio (tire content) significantly influences the overall silty sand strength (see Figure 10). At 100 kPa, the deviatoric stress of the silty sand without tire reinforcement (365.32 kPa) consistently exceeds that of all the tire-modified mixtures, with a 3% tire content exhibiting a moderate reduction, whereas the 6% and 9% mixtures, irrespective of tire size, demonstrate varying stress outcomes.

At 300 kPa, the effect of the tire composition becomes more significant, especially with the 3% and 9% tire blends. The 3% tire percentage, particularly the 5–10 mm size, results in the highest deviatoric stress (1259.7 kPa), surpassing those of the other combinations in this category. The 3% and 6% tire combinations produce elevated stresses, indicating that a greater tire percentage enhances the strength of the silty sand. The enhancement is not linear; the 6% tire mixture (across all sizes) frequently yields a lower deviatoric stress than the 3% tire mixture does, especially for the 5–10 mm and 10–20 mm sizes, where the values decrease markedly (1161.625 kPa and 906.18 kPa, respectively). This tendency indicates that beyond a specific tire percentage (e.g., 3%), an increase in the tire content may not universally enhance the silty sand strength and could be affected by the interplay between the tire particle size and the total mixture composition.

At 500 kPa, the deviatoric stresses reach their maximum values across all combinations, with the 3% tire mixture exhibiting a comparatively lower strength than the 6% and 9% mixtures. The 3% tire mixture, specifically the 5–10 mm size mixture, results in the highest deviatoric stress (2064.41 kPa), closely followed by the 6% tire (5–10 mm) and 9% tire (5–10 mm) mixtures, indicating that an increased tire content substantially improves the stress-bearing capacity of the silty sand under elevated confining pressure. The 6% tire combinations demonstrate somewhat reduced stresses at 500 kPa relative to those of the 3% and 9% tire mixtures, especially for larger tire diameters (10–20 mm), where the disparities in stress levels are more evident. This may suggest that when the tire content is above a specific level, the total mixture may become less efficient, especially with larger tire sizes that may not interlock as effectively with small tire particles.

On the basis of the average of all test findings (see Table 6), the CRT mixed with silty sand that has 5–10 mm of crumb at contents of 3%, 6%, and 9% has the highest deviatoric stress. Furthermore, the mixture containing 3% crumb tires 5–10 mm in size exhibited strong deviatoric stress, and the deviatoric stress was lowest for the mixture containing 6% crumb tires 3–6 mm in size. This study demonstrates the efficacy of crumb tires as a silty sand reinforcement material because they perform noticeably better in terms of deviatoric stress than pure silty sand.

### 4.4. Influence of the Confining Pressure

The three graphs (Figure 10) show the correlation between deviatoric stress (σ) and confining pressure (kPa) for varying percentages and dimensions of CRT mixed with silty sand. Each graph shows data for different percentages of tire particles, 3%, 6%, and 9%, along with their corresponding diameters (3–6 mm, 5–10 mm, and 10–20 mm, respectively). The overarching trend in all three figures indicates a rise in deviatoric stress with increasing confining pressure, which aligns with expectations in geotechnical engineering, as confinement augments soil strength. The impact of tire reinforcement at each pressure level is contingent upon the proportion and dimensions of the tire particles.

The incorporation of tire particles in the 3% tire reinforcement (Figure 10A) significantly elevates the deviatoric stress under elevated confining pressures of 300 kPa and 500 kPa. The effect is more pronounced for the 3% tire size of 5–10 mm than for the smaller sizes, where the deviatoric stress increases more gradually with increasing confining pressure. At a reduced confining pressure (100 kPa), the influence of tire reinforcement is inferior to that of unreinforced structures, with a deviatoric stress approximating that of pure silty sand. This finding indicates that tire particles may exert a greater impact when the silty sand experiences increased confinement.

In Figure 10B, with 6% tire reinforcement, the deviatoric stress increases with increasing confining pressure, and the disparity among the various tire sizes becomes more evident. The tire size of 5–10 mm appears to yield the largest deviatoric stress at 500 kPa, although the 3–6 mm and unreinforced variants exhibit comparable behavior at the same pressure. The prevailing trend demonstrates that an increased tire content (6% versus 3%) enhances the contribution of tire particles to silty sand strength, especially under elevated confining pressures, which is likely attributable to improved particle interlocking or frictional characteristics at greater compaction levels.

In Figure 10C, the impact of 9% tire reinforcement is markedly pronounced, as the deviatoric stress for all tire sizes considerably exceeds that of pure silt sand at elevated confining pressures. The tire sizes of 5–10 mm and 10–20 mm exhibit comparable stress levels at 500 kPa (refer to Figure 10C), indicating that at 9% tire content, size variation may exert a diminished influence on strength compared with smaller percentages. This tendency may suggest that the silty sand’s efficacy is increasingly influenced by the total volume of tire particles rather than their size after the tire content surpasses a specific level.

Generally, at a confining pressure of 100, the stress in reinforced silty sand is lower than that in unreinforced silty sand (refer to Figure 10A–C); however, at elevated confining pressures, the stress increases, but not to the same extent.

The findings show that the best size for improving the mechanical performance of silty sand is 5–10 mm crumb rubber particles, especially when the deviatoric stress response under various confining pressures is examined (see Figure 10 and Table 6). This implies that the size of the crumb rubber tire particles is a crucial factor in determining how the silty sand–rubber matrix behaves, preventing segregation or undue silty sand structure disruption. Additionally, this size makes it possible for the silty sand grains to better interlock mechanically and frictionally, which improves stress transmission under loading circumstances.

The particles are relatively small in the 3–6 mm range and may function more like fine inclusions, filling gaps with little reinforcement or interlocking. In addition to acting similarly to fine inclusions, these finer particles might not appreciably increase the load-bearing capacity and instead aid in damping rather than structural support. Therefore, there is a slight improvement in deviatoric stress.

Conversely, rubber particles that are 10–20 mm in size add heterogeneity to the mixture even though they are larger and might provide better interlock. Particularly at greater confining pressures, their size may result in zones of stress concentration and nonuniform stress distribution, which lower the overall strength and stiffness of the composite. Furthermore, when mixing, larger particles may separate, jeopardizing the consistency and uniformity of performance.

### 4.5. Influence of Triaxial Test Conditions

**Figure 11:**
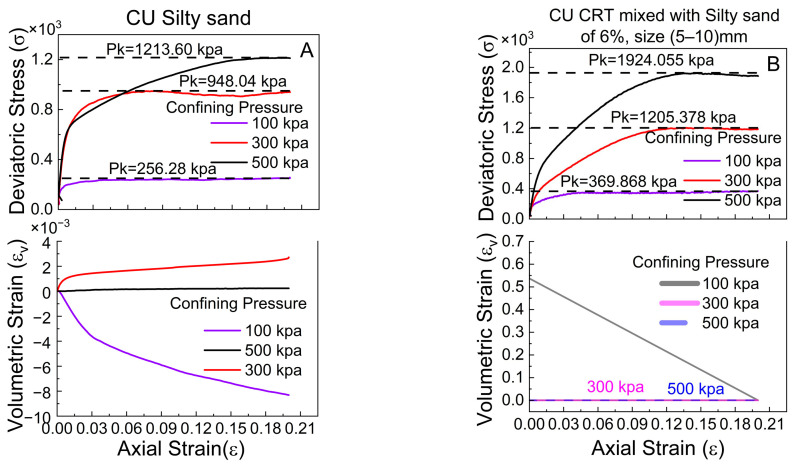

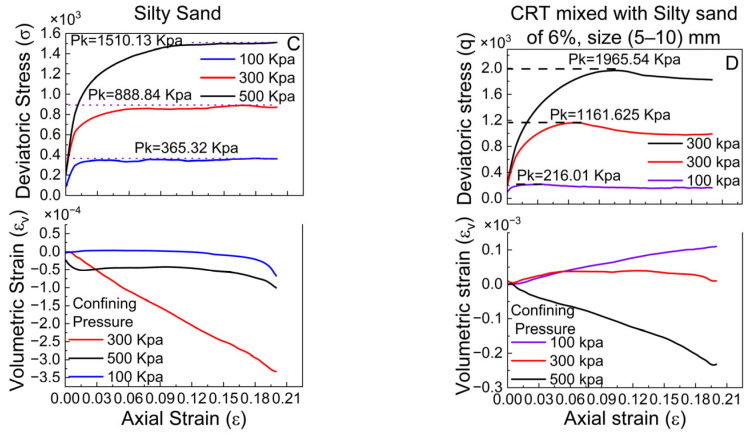
Deviatoric stress, volumetric strain, and axial strain comparison of CU and UU tests on silty sand and CRT mixed with silty at 6% ratio.

The four figures below display the results from triaxial tests performed under two distinct conditions: consolidated-undrained (CU) and unconsolidated-undrained (UU) tests on silty sand and silty sand mixed with 6% tire crumb particles (5–10 mm in size). The objective of this study was to analyze the influence of triaxial compression tests on reinforced and unreinforced soils subjected to confining pressures of 100, 300, and 500 kPa. (Figure 11A,B) are related to the CU test results, whereas (Figure 11C,D) are related to the UU test results. In the CU test, the silty sand sample is initially consolidated under confining pressure, facilitating the drainage of pore water, followed by shearing of the sample under undrained circumstances. Conversely, in the UU test, the material is subjected to loading without a consolidation phase.

The results for unreinforced silty sand indicate that in the CU test (Figure 11A), the deviatoric stress at failure (Pk) markedly increases with increasing confining pressure. The silty sand demonstrates strain-hardening characteristics, with peak stresses ranging from approximately 256 kPa at a confining pressure of 100 kPa to 1213 kPa at a confining pressure of 500 kPa. The volumetric strain graphs indicate a slight contraction phase at lower pressures, followed by a propensity for dilatation, particularly at elevated pressures. This signifies that the silty sand initially compacts under shear stress but subsequently expands due to particle rearrangement, which is characteristic of denser, well-consolidated materials. Conversely, the UU test (Figure 11D) results in elevated peak deviatoric stresses at identical confining pressures relative to those of the CU test, with a confining pressure of 100 kPa yielding a peak stress of approximately 365 kPa, markedly surpassing that of the CU test. The volumetric strain behavior varies, with the silty sand demonstrating continuous contraction (negative volumetric strain), particularly under elevated confining pressures of 300 kPa and 500 kPa (Figure 11A,B). This is characteristic of weaker, less cohesive silty sand structures that are not permitted to consolidate before shearing. The lack of a consolidation phase prevents silty sand particles from reorganizing into a more stable configuration, leading to elevated strength and increased volumetric strain (compression) during shear. This finding underscores the importance of pre-shear consolidation in enhancing silty sand strength.

The influence of triaxial test conditions on a reinforced silty sand mixture containing 6% crumb tires is shown in Figure 11B,C. In the CU test depicted in Figure 11B, the deviatoric stress markedly increased with axial strain, attaining peak values of 1924 kPa with an imposed confining pressure of 500 kPa. The volumetric strain response demonstrates initial contraction, which subsequently stabilizes, especially under elevated confining pressures. This trend indicates that the material experiences significant stress alterations due to draining during consolidation. Conversely, the UU condition (Figure 11C) results in reduced peak deviatoric stress values, especially at lower confining pressures of 100 and 300 kPa, whereas higher values are observed at 500 kPa (refer to Figure 11B,C). The volumetric strain trends at 500 kPa compress, whereas those at 100 and 300 kPa expand, with CUs remaining near zero or negative, signifying negligible volume changes due to the undrained characteristics of the test. The CU test offers lower peak strengths and facilitates a more accurate evaluation of long-term silty sand behavior, whereas the UU test offers insights into short-term stability under rapid loading conditions.

## 5. Determination of Triaxial Parameters

### 5.1. Expression Formula for Analysis of Data

An examination of triaxial test findings, encompassing both unconsolidated-undrained (UU) and consolidated-undrained (CU) tests, was performed to assess key parameters identified from the tested silty sand. The Mohr–Coulomb failure criterion served as the primary foundation for analyzing the test data. The *Hillslope Hydrology and Stability* book states the equation of shear stress [38] as follows:

(1) $$\tau_{max\;}=c+\sigma_{n}Tan\left(\theta \right)$$

This equation can be expressed in the principal stress criterion as follows:

(2) $$f\equiv \left(\sigma_{1}-\sigma_{3}\right)-\left(\sigma_{1}+\sigma_{3}\right)\sin\;\theta -2c\;\cos\;\theta =0$$

where (***τ***) expresses the shear stress; (***c***) represents the cohesion angle; (θ) represents the internal friction angle; ***σ*_1_** represents the maximum principal stress; and ***σ*_3_** represents the confining pressure applied in a triaxial test.

From the research paper published by *Sharafutdinov Rafael* [39], the stiffness modulus can be expressed as follows:

(3) $$E_{50}=\frac{\sigma_{1}-\sigma_{3}}{2}$$

This expression can be rewritten as follows:

(4) $$E_{50}=\frac{q_{max}}{2}$$

(***E*_50_**) is a secant stiffness modulus represented by the Young’s modulus (***E***), and $q_{max}$ is the maximum deviatoric stress.

These parameters are essential for understanding the mechanical behavior and strength characteristics of silty sand under different loading conditions. By plotting the Mohr–Coulomb circles and identifying the critical shear stress at failure, a strength envelope was established, providing insight into the shear resistance and deformation properties of the material. This approach allows for the comprehensive assessment of silty sand stability, which is crucial for evaluating its performance in engineering applications.

### 5.2. The Secant Stiffness Modulus

#### Unconsolidated-Undrained Stiffness Moduli of Silty Sand and CRT-Mixed with Silty Sand at 3%, 6%, and 9% Crumb Tires and Sizes Ranging from 5 to 10 mm

**Figure 12:**
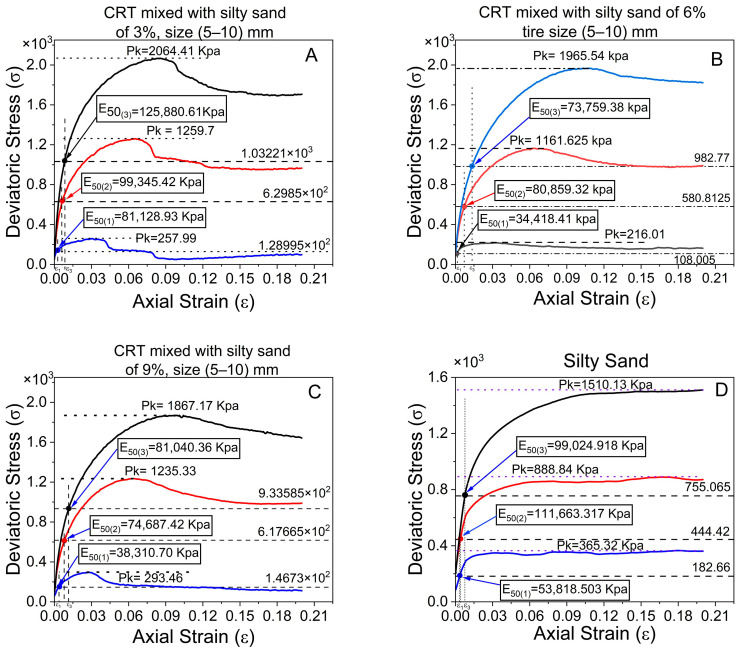
Determination and comparison of Stiffness modulus of silty sand and CRT mixed with silty sand at 3%, 6%, and 9%, with sizes ranging from 5 to 10 mm.

The secant stiffness, or E_50_, measures the characteristics of the deformation of the soil under applied stress (Figure 12). It displays the slope of the stress–strain curve at a point equal to 50% of the maximum deviator stress. This property is essential for comprehending the compressibility of silty sand materials. The comparative investigation of silty sand and CRT mixed with silty sand with sizes of 5–10 mm yields some intriguing results. The initial stiffness of silty sand combined with crumb tires at concentrations of 6% and 9% is lower than that of pure silty sand (Figure 12B,C) and Table 5, regardless of the maximal deviatoric stress to axial strain. This suggests that the resistance of the soil to deformation under initial loading circumstances decreases when a crumb tire is added at this level [40]. Because the crumb tire particles are compressible, they can absorb some of the applied load and cause more deformation, which is why the stiffness has decreased.

Interestingly, the average stiffness of silty sand containing 3% crumb tires differs from that of pure silty sand (Figure 11A,D and Table 7). This implies that the influence on the deformation behavior of the silty sand at a low ratio is less noticeable and that the intrinsic qualities of the silty sand take center stage at a reduced tire content. At ratios of 6% and 9%, the stiffness is less than that of pure silty sand. Generally, the stiffness modulus (E_50_) tends to decrease as the deviatoric stress increases. In soil mechanics, this phenomenon is frequently observed, where more stress causes more substantial deformation and a commensurate decrease in the stiffness of the soil [41] which is different in this observation, see Table 7.

**Figure 13:**
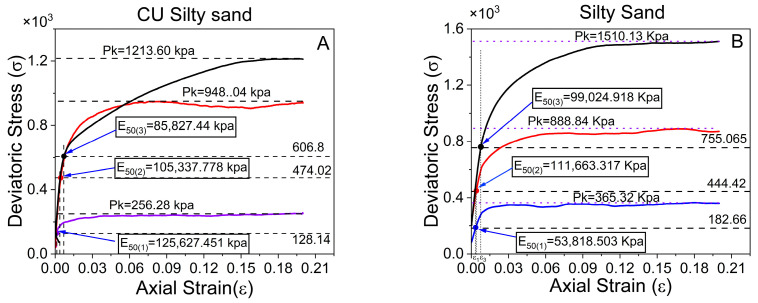

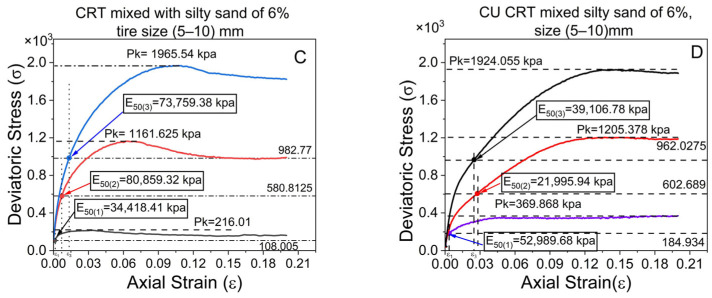
Comparison of the secant stiffness modulus of the CU and UU tests of silty sand and CRT mixed with silty sand at 6%, size 5–10 mm.

The comparison of the stiffness moduli of silty sand and CRT mixed with silty sand differs significantly between consolidated–undrained (CU) and undrained (UU) tests at various confining pressures (refer to Figure 13). At 100 kPa, the CU test has a 57% greater rigidity modulus (125,627.45 kPa) than the UU test does; however, at higher pressures (300 and 500 kPa), the CU findings slightly underperform the UU, even though the changes are minor (see Table 8 and Figure 13A,B). Considering that CRT is mixed with silty sand at 100 kPa, CU tests reveal a 35% (52,989.68 kPa) higher stiffness modulus than UU tests (refer to Table 9 and Figure 13B,C).

However, as the confining pressure increases, UU tests produce higher stiffness, most likely because of their faster, nonconsolidated nature. Similarly, Surarak et al. reported that the stiffness moduli derived from triaxial tests increase with increasing confining pressure [42]. Stiffness changes are explained by variations in strain amplitude rather than loading rates [43]. However, CU tests reflect more in situ conditions through full saturation and consolidation, albeit with potentially higher initial void ratios due to the absence of pre-shear consolidation. From Table 7, Table 8 and Table 9, the stiffness is not constant, contrary to the results reported by Balani et al., which decreases as the confining pressure increases [41]. Overall, the differences in the stiffness modulus between the two approaches are remarkable across pressure ranges.

From Figure 12 and Table 7 show that a stiffness modulus of 5–10 mm for CRT mixed with silty sand with 3%, 6%, and 9% ratios of 6% demonstrates optimal performance when the average results are considered. Additionally, Table 8 and Table 9 compare consolidated and unconsolidated-undrained tests, revealing that the CRT mixed with silty sand at sizes of 6% and 5–10 mm also exhibited superior performance.

Because of their high elasticity and low stiffness, crumb rubber particles add a soft compressible phase to the silty sand matrix. Rubber particles improve contact between silty sand grains and fill voids at lower contents (3%), marginally increasing ductility without significantly decreasing stiffness. At 6%, however, the rubber-to-silty-to-sand ratio reaches a point where the silty-sand skeleton is disturbed, decreasing the efficiency of silty-sand contact and diminishing the number of channels via which stress is transmitted. This lowers the overall stiffness modulus by making the matrix more deformable.

On the other hand, even when more rubber is present at 9%, the particles begin to interact and overlap, creating a secondary structure that may help with partial load-bearing capacity through energy absorption and rubber–rubber contact, which could account for stiffness stabilization or recovery.

Consequently, the 6% stiffness decline points to a crucial mixture transition point when the crumb rubber changes the silty sand structure in a way that reduces stiffness without yet offering sufficient material interaction to compensate for the loss. This highlights the necessity of more research into the ideal composition, particle interactions, and mechanisms of stress distribution in soils reinforced with rubber.

### 5.3. The Peak Strength

The impacts of the crumb rubber tire size, tire content ratio, and confining pressure on the peak stress are evident in the patterns presented in the provided data (see Table 6). As the confining pressure increases from 100 kPa to 500 kPa, the peak stress of all the combinations increases, demonstrating that greater confinement improves the strength of the silty sand tire mixtures. When various tire sizes are compared at identical ratios, larger tire particles (5–10 mm and 10–20 mm) generally exhibit elevated average peak stress values relative to those of smaller particles (3–6 mm), especially at 300 kPa and 500 kPa (see Figure 8). This finding indicates that larger CRT particles enhance mechanical interlocking and strengthening under elevated pressures, hence improving the load resistance.

The CRT content ratio significantly influences the peak stress. At CRT contents of 3%, 6%, and 9%, the peak stress values vary; nonetheless, there is a discernible trend indicating that a 9% CRT content yields elevated peak stresses, particularly under increased confining pressures of 300 kPa and 500 kPa. The 5–10 mm tire size distinctly demonstrates that the 3%, 6%, and 9% combinations exhibit average peak stresses over 1100 kPa, markedly surpassing the stress of silty sand alone (921.43 kPa). This suggests that appropriate confinement with increased CRT content, particularly in the mid-sized range, can augment the overall strength by enhancing particle interlocking and stress distribution.

### 5.4. The Shear Strength

**Figure 14:**
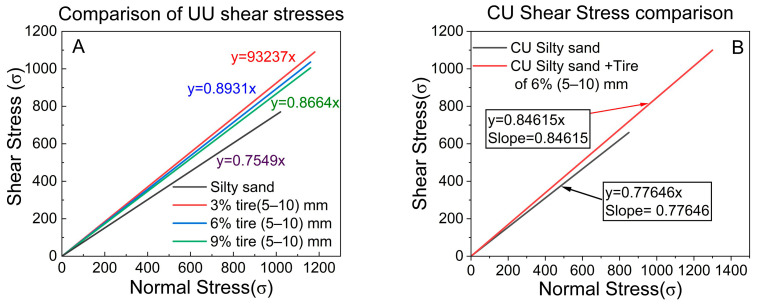
Comparison of the shear stresses of the unconsolidated and consolidated undrained tests of silty sand and CRT mixed with silty sand at 6%, size 5–10 mm.

In general, on the basis of the triaxial test results of the UU and CU tests on CRT mixed with silty sand at a 6% crumb tire, a size of 5–10 mm, and pure silty sand (see Table 10 and Table 11, Figure 14), the CU provides a high friction angle of 37.8 degrees greater than 0.8 UU (Figure 14A,B), which implies that the higher the friction angle is, the higher the shear stress failure line (Table 10 and Table 11). However, on CRT mixed with silty sand, the UU provides higher shear stress because its friction angle of 41.7 degrees is greater than that of the 1.5 degree, which is 40.2 degrees in the CU tests. For CRT mixed with silty sand, Youwei Xu et al. reported that wet tests consistently yield lower shear strengths than dry tests do, leading to diminished apparent cohesions and friction angles [44].

The shear stress analysis was performed on the following mixtures at ratios of 3%, 6%, and 9%: silty sand and CRT mixed with silty sand with (5–10) mm sizes, concerning the unconsolidated–undrained UU triaxial tests. The CRT mixed with silty sand (5–10 mm) with a 3% ratio resulted in the highest deviatoric stress among the samples under study (Table 10 and Figure 14A).

Alongside the deviatoric stress, the shear stress increased, which automatically affected the friction angle in the following ways: silty sand (Φ = 37.0°), CRT mixed with silty sand at 3% (Φ = 42.9°), 6% (Φ = 41.7°), and 9% (Φ = 40.9°) had the lowest degree of friction (refer to Table 10). The results indicate that the friction angle increased when CRT was added to the silty sand as opposed to when only silty sand was used. LDPE-to-sand particle connections, on the other hand, have lower frictional properties than do sand-to-sand particle contacts because of soil particle rearrangement [41].

This also means that the addition of CRT mixed with silty sand enhances the frictional properties and shear strength of silty sand. The highest friction angle was demonstrated by the silty sand mixture with 3% crumb rubber tires, suggesting that this ratio provides the best balance between the benefits of the CRT and the overall soil attributes. While it remained higher than that of the silty sand mixture alone, the friction angle gradually decreased as the CRT content increased to 3%, 6%, and 9%. These findings suggest that a variety of CRT compositions are perfect for improving the shear strength and frictional qualities of soil [45].

In summary, precisely identifying the essential parameters for reinforcing silty sand with crumb tire components is vital for attaining optimal performance. The findings of this study indicate that peak strength is crucial in influencing the maximum deviatoric stress applied in triaxial tests on reinforced silty sand. Shear stress dictates the failure characteristics of reinforced silty sand, affecting its resistance capability and aiding in the determination of the internal friction angle and cohesiveness. The stiffness modulus characterizes the interaction between silty sand and crumb tire particles, indicating an improvement in stiffness resulting from compromised interconnection bonds.

The use of the following formula to effectively address and optimize the parameters of reinforced CRT mixed with silty sand can be helpful.

The deviatoric stress is expressed as follows:

(5) $$q=\frac{F}{A}$$

which can also be expressed as follows:

(6) $$q_{max}=\frac{F_{max}}{A}=\sigma_{1}-\sigma_{3}$$

The strain can be calculated as follows:

(7) $$\varepsilon =\frac{∆l}{l}$$

The internal friction angle is expressed as follows:

(8) $$\tan\emptyset =\frac{\sigma_{1}-\sigma_{3}}{2c+(\sigma_{1}+\sigma_{3})}$$

If c=0, then

(9) $$\tan\emptyset =\frac{{(\sigma }_{1}-\sigma_{3})}{\left(\sigma_{1}+\sigma_{3}\right)}$$

where (∅) represents the internal friction angle; (***c***) denotes the cohesion angle; (***F***) signifies the axial load; (***A***) represents the area of the triaxial test where the axial load is applied; ***σ*_1_** represents the major principal stress; and ***σ*_3_** represents the minor principal stress (confining pressure).

## 6. Conclusions

After performing all the tests planned for the triaxial tests of CU and UU under different conditions and analyzing the results for silty sand and CRT mixed with silty sand with sizes of 3–6, 5–10, and 10–20 mm at 3%, 6%, and 9% crumb contents at 100, 300, and 500 kPa confining pressures, the results reveal that incorporating CRT into silty sand provides a promising and innovative solution for silty sand stabilization, increasing the deviatoric stress, shear stress, and friction angle and reducing the stiffness modulus to enhance both environmental sustainability and engineering performance. The following keys are found:

In general, triaxial tests under unconsolidated–undrained (UU) conditions reveal that the average deviatoric stress of CRT mixed with silty sand increases, except for combinations containing 3% and 6% crumb rubber at a size of 3–6 mm, where the deviatoric stress is lower than that of silty sand. The maximum deviatoric stress of 1194.033 kPa was observed in CRT mixed with 3% silty sand at a particle size of 5–10 mm. The influence results under the same confining pressure, consistent ratios, and various tire sizes indicate that the addition of silty sand with crumb tire sizes (5–10) at ratios of 3%, 6%, and 9% yields the greatest overall outcomes. An increase in deviatoric stress immediately indicates an increase in shear stress, which correlates with the friction angle of the silty sand.

The influence of shear stress on the triaxial test conditions of silty sand from the UU and CU indicates that the shear stress in the UU tests is lower than that in the CU tests. Conversely, with CRT mixed with silty sand containing 6% crumb tires (5–10 mm), the shear stress in the UU tests exceeds that in the CU tests. The stiffness modulus of the CU CRT mixed with silty sand at 6% and a tire size of 5–10 mm is inferior to that of the UU CRT mixed with silty sand with the same composition.

Following an exhaustive analysis of critical parameters derived from triaxial tests, such as deviatoric stress, confining pressure effects, crumb tire dimensions and proportions, peak strength, shear stress, and stiffness modulus, it is advised to utilise crumb tire particles measuring 5–10 mm in size with an optimal content of 6% in silty sand. This combination resulted in the most significant enhancement in the mechanical properties of CRT mixed with silty sand, increasing its shear strength and reducing stiffness due to interlocking while preserving advantageous stress–strain behavior.

## Figures and Tables

**Table 1:** Physical properties of the silty sand.

Soil Type	Density kg/m^3^	Moisture Content %	Internal Friction Angle	Cohesion Angle	Permeability cm/s
Silt sand	1580	27.45	37.04	0	1.54 × 10^−5^

**Table 2:** Chemical structure of silty sand.

N	Name	Chemical Structure	Function
1	Quartz	SiO_2_	Is the most prevalent mineral in sandy soils. It is chemically inert, stable, and weather-resistant, making it a key framework component.
2	Feldspar	KAlSi_3_O_8_-NaAlSi_3_O_8_-CaAl_2_Si_2_O_8_	It is a series of aluminum silicate minerals that are less durable than quartz; they are prone to chemical weathering and can convert into clay minerals over time.
3	Mica, also known as muscovite	KAl_2_(AlSi_3_O_10_)(OH)_2_	A sheet silicate mineral occurring in small particles helps to maintain the flexibility and cohesion of silty and clayey soil fractions.
4	Clay minerals, including kaolinite	Al_2_Si_2_O_5_(OH)_4_	Affect soil swelling, plasticity, and cation exchange capacity.

**Table 3:** Chemical structure of crumb tire.

*n*	Name	Chemical Structure	Function
1	Natural rubber (cis-1,4-polyisoprene)	-(C_5_H_8_) *n*-.	Allows for chemical crosslinking during vulcanization, increasing flexibility and robustness.
2	Styrene-butadiene rubber (SBR):	(C_8_H_8_), and (C_4_H_6_)	This copolymer has higher abrasion resistance and ageing stability than natural rubber, hence it is commonly utilized in tire manufacture.
3	Carbon black	c	It is used as a reinforcing filler. It improves the rubber’s mechanical strength, wear resistance, and UV stability.

**Table 4:** Unconsolidated––undrained specimen tests.

		1. Tire Size (3–6) mm		
TEST TYPE	Name	% of Tires	Confining Pressure	Number of Tests
UU	Silty sand	0%	(100 kpa,300 kpa and 500 kpa)	3
	CRT mixed with silty sand	3%	(100 kpa,300 kpa and 500 kpa)	3
	CRT mixed with silty sand	6%	(100 kpa, 300 kpa and 500 kpa)	3
	CRT mixed with silty sand	9%	(100 kpa, 300 kpa and 500 kpa)	3
		**2. Tire Size (5–10) mm**		
**TEST TYPE**	**Name**	**% of Tires**	**Confining Pressure**	**Number of Tests**
UU	CRT mixed with silty sand	3%	(100 kpa, 300 kpa and 500 kpa)	3
	CRT mixed with silty sand	6%	(100 kpa, 300 kpa and 500 kpa)	3
	CRT mixed with silty sand	9%	(100 kpa, 300 kpa and 500 kpa)	3
		**3. Tire size (10–20) mm**		
**TEST TYPE**	**Name**	**% of Tires**	**Confining Pressure**	**Number of Tests**
UU	CRT mixed with silty sand	3%	(100 kpa, 300 kpa and 500 kpa)	3
	CRT mixed with silty sand	6%	(100 kpa, 300 kpa and 500 kpa)	3
	CRT mixed with silty sand	9%	(100 kpa, 300 kpa and 500 kpa)	3

**Table 5:** Consolidated–undrained specimen tests.

		Tire Size (5–10) mm		
TEST TYPE	Name	% of Tires	Confining Pressure	Number of Tests
CU	CRT mixed with silty sand	0%	(100 kpa,300 kpa and 500 kpa)	3
	CRT mixed with silty sand	6%	(100 kpa, 300 kpa and 500 kpa)	3

**Table 6:** Average test results for deviatoric stress.

Soil Type	Applied Confining Pressure			Average (kPa)
	100 kPa	300 kPa	500 kPa	
**Silt Sand**	365.32	888.84	1510.13	921.430
3% Tire, size (3–6) mm	290.444	888.88	1557.53	912.285
6% Tire, size (3–6) mm	106.8	908.02	1502.8	839.207
9% Tire, size (3–6) mm	307.67	1136.64	1905.5	1116.603
3% Tire, size (5–10) mm	257.99	1259.7	2064.41	1194.033
6% Tire, size (5–10) mm	216.01	1161.625	1965.54	1114.392
9% Tire, size (5–10) mm	293.46	1235.33	1867.17	1131.987
3% Tire, size (10–20) mm	209.81	939.02	1674.32	941.050
6% Tire, size (10–20) mm	297.08	906.18	1809.02	1004.093
9% Tire, size (10–20) mm	246.89	1045.29	1686.56	992.913

**Table 7:** Average stiffness moduli of CRT mixed with silty sand at 3%, 6%, and 9%, with sizes ranging from 5 to 10 mm.

Stiffness at Cell Pressure	Silty Sand	CRT Mixed with Silty Sand at 3%	CRT Mixed with Silty Sand at 6%	CRT Mixed with Silty Sand at 9%
E_50_ at 100 kPa	53,818.5	81,128.93	34,418.41	38,310.7
E_50_ at 300 kPa	111,663.317	99,345.42	80,859.32	74,687.4
E_50_ at 500 kPa	99,024.91	125,880.61	73,759.38	81,040.36
Average	88,168.9	10,2118.32	63,012.37	64,679.48

**Table 8:** Stiffness moduli of CU and UU of silty sand.

Soil Type	E_50_ at 100 kPa	E_50_ at 300 kPa	E_50_ at 500 kPa	Average
UU Silty sand	53,818.5	111,663.317	99,024.918	88,168.9
CU Silty sand	125,627.45	105,337.77	85,827.4	105,597.54

**Table 9:** Stiffness modulus of CU and UU of CRT mixed with silty sand at 6%.

Soil Type	E_50_ at 100 kPa	E_50_ at 300 kPa	E_50_ at 500 kPa	Average
UU CRT mixed with silty sand 6%	34,418.41	80,859.32	73,759.38	63,012.37
CU CRT mixed with silty sand 6%	52,989.68	21,995.94	39,106.78	38,030.80

**Table 10:** UU cohesion and friction angle of silty sand and CRT mixed with silty sand.

Soil Type	Cohesion Angle (*c*)	Friction Angle (∅)
UU Silty sand	0	37.0
UU CRT mixed with silty sand at 3%	0	42.9
UU CRT mixed with silty sand at 6%	0	41.7
UU CRT mixed with silty sand at 9%	0	40.9

**Table 11:** CU cohesion and friction angle of CRT mixed with silty sand and unreinforced silty sand.

Soil Type	Cohesion Angle (*c*)	Friction Angle (∅)
CU silty sand	0	37.8
CU CRT mixed with silty sand at 6%	0	40.2

## Data Availability

The authors do not have permission to share data.
